# Protective Effects of Phosphocreatine Administered Post-Treatment Combined With Ischemic Post-Conditioning on Rat Hearts With Myocardial Ischemia/Reperfusion Injury

**DOI:** 10.14740/jocmr2087w

**Published:** 2015-02-09

**Authors:** Wenhua Zhang, Huizhen Zhang, Yanqiu Xing

**Affiliations:** a Qilu Hospital of Shandong University, Jinan 250012, China; b These authors contributed equally to this article.

**Keywords:** Ischemic post-conditioning, Myocardial reperfusion injury, Apoptosis, Phosphocreatine, TNF-α

## Abstract

**Background:**

The aim of the study was to investigate the effects of phosphocreatine (PCr) post-treatment combined with ischemic post-conditioning on myocardial ischemia/reperfusion (I/R) injury in a rat model.

**Methods:**

Forty Sprague-Dawley rats that had undergone 30 minutes ischemia and 120 minutes reperfusion were randomly divided into four groups (n = 10 in each group): the I/R group, the ischemia post-conditioning (IPost) group, the PCr group, and the IPost + PCr group. The activities of serum creatine kinase (CK), myeloperoxidase (MPO), and lactate dehydrogenase (LDH) were measured after 120 minutes of reperfusion. At the end of the experiment, serum levels of nuclear factor (NF)-κB and tumor necrosis factor (TNF)-α were detected, myocardial infarct size (IS) was measured by triphenyltetrazolium chloride staining, and myocardial expression of Bcl-2 and phosphorylated Akt (p-Akt) was determined by western blot.

**Results:**

The IPost, PCr, and PCr + IPost groups had significantly lower IS than the I/R group (P < 0.05). The reductions in CK, LDH, and MPO release were consistent with the decrease in the myocardial IS (P < 0.05). Serum concentrations of TNF-α and NF-κB in the IPost, PCr, and PCr + IPost groups were significantly lower than those in the I/R group (P < 0.05). The levels of p-Akt and Bcl-2 in the IPost, PCr, and PCr + IPost groups were greater than those in the I/R group (P < 0.05). CK, LDH, MPO, NF-κB, TNF-α, p-Akt, Bcl-2 and IS were further enhanced in the IPost + PCr group (P < 0.05).

**Conclusions:**

Post-treatment with PCr enhanced the protective effect of IPost on rat myocardium affected by I/R injury, possibly by inhibiting the inflammatory response and activating the phosphatidylinositol 3-kinase (PI-3K)/Akt/Bcl-2 signaling pathway.

## Introduction

Acute myocardial infarction (AMI) threatens human health and causes millions of deaths worldwide. It is the main cause of chronic heart failure, with a mortality rate of about 10% [1]. At present, early opening of the culprit vessel remains the most effective treatment method for acute myocardial injury. However, the resulting reperfusion injury cannot be ignored, as it has serious affects. Lethal myocardial injury, caused by ischemia/reperfusion (I/R), is known to comprise up to 50% of the final infarct zone of a myocardial infarct [2]. Accumulation of polymorphonuclear neutrophils and reactive oxygen species, vascular endothelial dysfunction, and high levels of cell apoptosis, combined with other factors, lead to serious injury and dysfunction of the myocardial tissue during early reperfusion. Ischemia post-conditioning (IPost) is a novel therapeutic method that can effectively reduce myocardial reperfusion injury. However, a potential drawback is the impairment of intracellular ATP generation due to repeated ischemia during reperfusion [3]. Phosphocreatine (PCr) is a high-energy phosphate compound, which serves as an ATP buffer, and it often effectively plays a protective role in I/R-injured myocardial tissue. For acute myocardial ischemia, application of exogenous creatine phosphate can reduce the release of cardiac enzymes, reduce myocardial infarct size (IS), reduce ventricular arrhythmias, prevent the enlargement of the ventricular chamber, and improve ventricular remodeling, thus significantly improving heart function. However, the mechanism by which it protects ischemia-injured myocardial tissue is not entirely clear. This study aimed to investigate the combined effects of PCr post-conditioning and IPost on myocardial I/R injury in a rat model and to study the mechanism of action in terms of the levels of proinflammatory cytokines and the apoptotic pathway.

## Materials and Methods

### Animals

All experimental procedures were performed in accordance with the guide of Shandong University for the use and care of laboratory animals. All surgical procedures were approved by the committee for experimental animals of the Centre for Disease Prevention and Control of Shandong Province. Forty male Sprague-Dawley rats weighing 260 - 290 g were acquired from the Experimental Animal Center of Shandong University, Jinan, China. The animals were maintained under standard laboratory conditions at 22 ± 2 °C and a relative humidity of 55±5%.

### Reagents

Sodium creatine phosphate was purchased from Alfa Wassermann S.P.A (Pescara, Italy). Antibodies against phosphorylated Akt (p-Akt), Bcl-2, and β-actin were obtained from Cell Signaling Technology (Beverly, MA). The bicinchoninic acid protein assay kit was obtained from Beyotime Institute of Biotechnology (Jiangsu, China). The creatine kinase (CK), lactate dehydrogenase (LDH), and myeloperoxidase (MPO) kits were obtained from Nanjing Jiancheng Bio Co. (Nanjing, China). The tumor necrosis factor (TNF)-α ELISA kit was purchased from Wuhan Eiaab Science Co. (Wuhan, China) and the nuclear factor (NF)-κB ELISA kit, from Blue Gene (Shanghai, China). Triphenyltetrazolium chloride (TTC) and Evans blue were supplied by Sigma Chemicals (St. Louis, MO).

### Experimental protocol

Establishment of a myocardial I/R model: Rats were anesthetized with an intraperitoneal injection of pentobarbital sodium (30 mg/kg). After endotracheal intubation, ventilation was provided via a rodent respirator at a respiratory rate of 60 times per minute with a tidal volume of 20 mL/kg [4]. A left parasternal incision was made through the third and fourth ribs, and the pericardium was then gently opened to expose the heart. The left lateral anterior descending artery (LAD) was ligated using a 7-0 silk suture 2 mm below the left atrial appendage and the left edge of the pulmonary cone. In addition, a medical latex tube (inner diameter, 1.5 mm) was placed between the ligature and the LAD. Myocardial ischemia was induced through compression of the LAD by tightening a silk suture around the latex tube. After 30 min of ischemia, the latex tube was removed to reperfuse the myocardium. Left ventricular (LV) anterior wall myocardial cyanosis and ECG standard lead II ST-segment elevation indicated the successful production of the model.

Heparin sodium (300 U/kg) was injected through the tail vein before coronary artery occlusion. Next, 40 Wistar rats were randomly divided into four groups (n = 10 in each group), and the LAD was occluded for 30 min and then reperfused for 120 min in these rats, as detailed above. 1) The I/R group was subjected to 30 min of ischemia followed by a 2-h reperfusion without other interventions. 2) The IPost group was subjected to three cycles of 10-s coronary occlusions, separated by 10-s reperfusions, before a 2-h reperfusion. 3) For the PCr group, PCr (200 mg/kg) was injected slowly through the femoral artery 5 min before reperfusion. Finally, 4) for the IPost + PCr group, IPost was applied firstly as above, and then PCr at 200 mg/kg was applied before the 2-h reperfusion. The I/R and IPost groups were administered the same amount of saline. After the 2-h reperfusion, blood was extracted from the femoral vein for serological testing. The myocardial IS was measured using TTC staining. Parts of the anterior wall of the LV myocardium were isolated for western blot analysis. Rats that developed intractable ventricular fibrillation were excluded from subsequent analysis.

### Measurement of CK, MPO, and LDH release

To test for CK, MPO, and LDH activity, 2 mL of femoral vein blood was drawn and centrifuged at 3,000 rpm for 15 min. The samples were analyzed by colorimetry according to the manufacturer’s instructions. The activities of these enzymes were expressed in U/L.

### Determination of the LV area at risk (AAR) and IS

At the end of the perfusion protocols, the coronary artery was reoccluded and 2 - 3 mL of 1% Evans blue dye was injected into the carotid artery to distinguish between the perfused and non-perfused (AAR) heart sections. Stained hearts were frozen at -20 °C for 10 min and then sliced into four or five 2-mm cross-sections across the LV long axis. The sections were then incubated at 37 °C for 20 min with 1% TTC to delineate the infarcted tissue, and then fixed for 30 min in 10% formaldehyde. The AAR of the infarction was colored red while the infarcted area (IA) within the AAR remained pale yellow. The AAR myocardium and IA myocardium were carefully separated under an optical microscope. The separated myocardial tissues were weighed and the myocardial ischemic area was expressed as AAR/LV, while the myocardial IS was expressed as IA/AAR [5].

### Serum concentrations of NF-κB and TNF-α

Blood was collected 120 min after I/R and the serum was extracted. The concentrations of TNF-α and NF-κB in the serum were determined using the respective ELISA kits according to the manufacturers’ instructions.

### Western blotting analysis of p-Akt and Bcl-2

LV anterior ischemic myocardial tissues were obtained 120 min after reperfusion for detection of p-Akt and Bcl-2 proteins. The myocardial tissues were pulverized and dissolved in lysis buffer. The solution was then homogenized and centrifuged at 14,000 g for 15 min at 4 °C. The supernatant was resolved on a 10% SDS-polyacrylamide gel and transferred to nitrocellulose membranes. After the membranes were blocked with Tris-buffered saline-Tween 20 (TBS-T, 0.1% Tween 20) containing 5% non-fat dried milk for 2 h at room temperature, they were washed three times with TBS-T and incubated overnight at 4 °C with the following primary antibodies: rabbit polyclonal anti-p-Akt (at Ser^473^; 1:300 dilution) and anti-Bcl-2 (1:500 dilution). β-Actin (1:1,000 dilution) was used as a standard control for protein quantity. The membranes were then washed three times with TBS-T for 15 min and incubated for 2 h at room temperature with horseradish peroxidase (HRP)-conjugated secondary antibodies at dilutions of 1:5,000, 1:10,000 and 1:20,000. After extensive washing, immune complexes were detected using an infrared imaging system. Gray-scale ratios of p-Akt/β-actin and Bcl-2/β-actin were calculated using ImageJ Software and used as semi-quantitative indicators of protein expression levels.

### Statistical analysis

Data were expressed as mean ± SD and analyzed with SPSS16.0 statistical software. Data were compared between groups using one-way analysis of variance. Statistical significance was set at P < 0.05.

## Results

### Effects on CK, LDH, and MPO release and myocardial IS

The measurements of CK, LDH, and MPO release and the myocardial IS are shown in Tables 1 and 2, respectively. After 120 min of reperfusion, no significant difference was found in the myocardial ischemic area (AAR/LV) between the I/R group, IPost group, and PCr group, but the PCr + IPost group showed significantly reduced AAR/LV compared to the remaining groups. The IPost and PCr groups had a significantly lower IS than the I/R group, and the PCr + IPost group had an even lower IS. The reductions in CK, LDH, and MPO release were consistent with the decrease in the myocardial IS.

**Table 1:** Comparison of Serum CK, LDH, and MPO in Each Experimental Group (x ± s, n = 10).

Group	CK (U/L)	LDH (× 10^3^ U/L)	MPO (U/L)
I/R	16.42 ± 2.74	5.21 ± 0.34	73.16 ± 8.41
IPost	8.74 ± 1.19*	2.90 ± 0.14*	37.48 ± 6.36*
PCr	9.04 ± 1.42*	2.80 ± 0.12*	36.65 ± 3.71*
PCr + IPost	5.79 ± 1.34*†	1.85 ± 0.15*†	19.92 ± 4.16*†

*P < 0.05 vs. I/R group; †P < 0.05 vs. IPost group and PCr group.

**Table 2:** Percentages of Risk Area and Infarct Size (x ± s, n = 7)

Group	BW (g)	LV (g)	AAR/LV (%)	IS (%)
I/R	278.24 ± 3.34	0.79 ± 0.02	36.74 ± 5.13	51.04 ± 3.88
IPost	280.83 ± 11.12	0.80 ± 0.05	35.62 ± 3.22	38.27 ± 2.67*
PCr	279.48 ± 6.79	0.79 ± 0.06	35.40 ± 4.18	35.78 ± 3.57*
PCr + IPost	275.20 ± 8.56	0.78 ± 0.03	24.85 ± 3.10*†	18.52 ± 1.33*†

*P < 0.05 vs. I/R group; †P < 0.05 vs. IPost group and PCr group. BW: body weight; LV: left ventricle.

### Expression of TNF-α and NF-κB following I/R injury

Serum concentrations of TNF-α and NF-κB were measured after I/R injury in all four experimental groups (Table 3). Serum concentrations of TNF-α and NF-κB in the IPost, PCr, and PCr + IPost groups were significantly lower than those in the I/R group, and the concentrations in the PCr + IPost group were significantly lower than those in the IPost and PCr groups (P < 0.05).

**Table 3:** Comparison of Serum NF-κB and TNF-α (x ± s, n = 7)

Group	NF-κB (ng/mL)	TNF-α (pg/mL)
I/R	67.98 ± 8.22	34.95 ± 5.12
IPost	40.92 ± 4.47*	17.46 ± 1.97*
PCr	16.81 ± 2.17*	39.74 ± 2.77*
PCr + IPost	26.23 ± 2.68*†	10.83 ± 1.63*†

*P < 0.05 vs. I/R group; †P < 0.05 vs. IPost group and PCr group.

### Effects on the expression of p-Akt and Bcl-2

**Figure 1:**
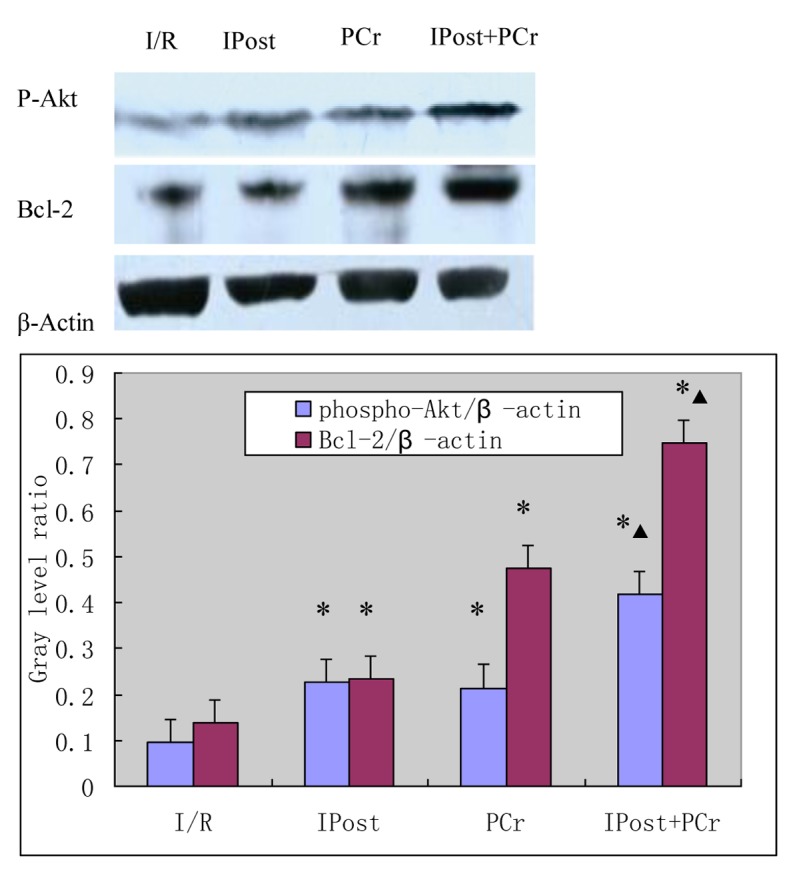
Results of western blot detection of left ventricular anterior ischemic myocardium p-Akt and Bcl-2 protein expression. The corresponding densitometric analysis is shown as a bar chart. *P < 0.05 vs. I/R group; ▲P < 0.05 vs. IPost group and PCr group.

As shown in Figure 1, expression of p-Akt at Ser473 and Bcl-2 was measured by western blot at 120 min after reperfusion. The levels of p-Akt and Bcl-2 in the IPost, PCr, and PCr + IPost groups were greater than those in the I/R group (p-Akt, 0.228 ± 0.012, 0.214 ± 0.011, 0.418 ± 0.012 vs. 0.094 ± 0.009; Bcl-2, 0.234 ± 0.012, 0.475 ± 0.013, 0.749 ± 0.016 vs. 0.139 ± 0.011; P < 0.05). The levels of p-Akt and Bcl-2 in the PCr + IPost group were higher than those in the IPost and PCr groups (P < 0.05). The protein levels for each sample were determined as a percentage of the corresponding β-actin levels.

## Discussion

Despite the recent advances in cardiovascular research, ischemic heart disease remains the most common cause of death worldwide [6]. Further, reperfusion therapy, which promotes the rapid recovery of blood flow to the myocardial ischemic zone, may result in further complications such as diminished cardiac contractile function and irreversible tissue necrosis, which are collectively known as I/R injury [7]. It has been argued that lethal myocardial injury caused by I/R constitutes up to 50% of the final IS [2]. Therefore, reducing reperfusion injury has become one of the focuses of clinical research. IPost is a series of transient mechanical interruptions of reperfusion at its very onset [8]. Because IPost can be more tightly controlled clinically and is more predictable than IPost, it may be a promising approach to limit IS during the revascularization of patients with AMI [9]. Lonborg et al reported that intervention with IPost resulted in a significant reduction of myocardial enzyme release and improved LV function [10].

PCr is a very important energy substrate and has the dual function of storing and transporting ATP in energy metabolism. Exogenous PCr provides energy directly to the cell through the PCr/CK system. The pharmacokinetics of PCr shows a biphasic distribution in the blood, with clearance half-times of 7 and 50 min after injection of a single dose. Although the clearance rate is initially rapid, PCr is specifically absorbed by the heart due to its high affinity to the mycardium. Therefore, because of this direct interaction, exogenous PCr may play an important role in the ischemic myocardium. Prabhakar et al reported that during periods of myocardial ischemia in rats, preconditioning with PCr could improve the levels of intracellular ATP and attenuate metabolic stress [11]. Because drug post-conditioning and IPost are better prospects for clinical application than preconditioning, we first sought to establish PCr post-conditioning animal models by administering a large dose of PCr (200 mg/kg). In this study, we demonstrated that IPost can reduce serum CK and LDH levels as well as myocardial IS. PCr combined with IPost can further reduce the levels of serum CK and LDH and the myocardial IS, indicating that PCr post-conditioning strengthens the role of IPost in myocardial protection. Further, the combination has a better protective effect on I/R-damaged myocardial tissue than either treatment alone.

In reperfusion injury, mitochondrial damage may cause a loss of cardiomyocyte function and viability. The major mechanism of mitochondrial dysfunction may be the extended opening of the mitochondrial permeability transition pore (mPTP) [1]. This extended opening of the mPTP may induce cytochrome C release, leading to the formation of apoptotic bodies and resulting in apoptosis. Apoptosis is a regulated form of cell death, and inhibiting cardiomyocyte apoptosis has become an important area of research. The phosphatidylinositol 3-kinase (PI-3K)/protein kinase B (Akt, also known as PKB)/Bcl-2 pathway regulates a diverse set of cell activities, including the survival and apoptosis of many different cell types [12, 13]. When upstream signaling stimulates the cell surface G-protein coupled receptors, PI-3K activation promotes phosphorylation of Akt at Ser^473^ and Thr^308^, which activates the downstream protein Bcl-2 and has a cardio-protective effect. The Bcl-2 family of proteins plays an important role in regulating apoptosis in the heart. Bcl-2 has been reported to inhibit activation of Bax/Bak to prevent permeabilization of the outer mitochondrial membrane, and it blocks the opening of the mPTP to improve the calcium threshold in heart mitochondria. In addition, the number of apoptotic cells in the infarct zone was found to be reduced and recovery of cardiac function improved after I/R in transgenic mice overexpressing Bcl-2 in the heart [14]. In our study, we measured the expression of p-Akt Ser^473^ and Bcl-2 by western blot. The levels 2 in the IPost, PCr, and PCr + IPost groups were greater than those in the I/R group. PCr post-conditioning, in addition to IPost, significantly increased protein expression of p-Akt and Bcl-2 in myocardial tissue and had a better protective effect on myocardial I/R injury than either treatment alone. Finally, the myocardial IS was significantly reduced. With regard to the channels that IPost combined with PCr post-conditioning may use to promote PI-3K activation, we speculate that this may be related to the co-activation of cell surface adenosine receptors. Because exogenous PCr can be quickly converted into ATP, and ATP’s final decomposition product is adenosine, IPost could also contribute to activation of cell surface adenosine receptors. This could be further confirmed by experimentation with an adenosine receptor blocker.

Along with I/R, the inflammatory response also aggravates myocardial injury [15]. Myocardial infarction induces neutrophil infiltration into the infarct zone, and this infiltration can lead to myocardial damage [16]. Since the enzyme MPO is mainly released from neutrophils, the MPO activity assay has became widely used as a biomarker for inflammatory reactivity and neutrophil infiltration. In our study, PCr post-conditioning combined with IPost could significantly attenuate MPO activity, and this exerted a myocardial protective effect by inhibiting the inflammatory process. The inflammatory response during I/R injury is exacerbated by production of inflammatory mediators (e.g., TNF-α and IL-1β) by neutrophils [17]. Further, the TNF-α and IL-1β released from the I/R-injured myocardium can activate NF-κB, which quickly enters the nucleus and initiates expression of inflammatory factors, which then accelerate the inflammatory reaction and form positive feedback [18]. In our study, the significant reduction of TNF-α and NF-κB by PCr post-conditioning and IPost suggested the possible myocardial protective effect of these treatments through the inhibition of proinflammatory cytokine release.

### Conclusion

PCr post-conditioning combined with IPost reduced the levels of serum CK and LDH and the myocardial IS in an *in vivo* rat heart model. These protective mechanisms may be partly related to the activation of the PI-3K/Akt/Bcl-2 signaling pathway and inhibition of the inflammatory response. Because PCr post-conditioning can significantly strengthen the cardio-protective effects of IPost, it may offer new treatment methods and further ideas for the prevention of clinical reperfusion injury.
